# Direct reprogramming and biomaterials for controlling cell fate

**DOI:** 10.1186/s40824-016-0086-y

**Published:** 2016-12-07

**Authors:** Eunsol Kim, Giyoong Tae

**Affiliations:** School of Materials Science and Engineering, Gwangju Institute of Science and Technology, Gwangju, 61005 Republic of Korea

**Keywords:** Direct reprogramming, Stem cell, Surface, Growth factors, ECM, Gene delivery

## Abstract

Direct reprogramming which changes the fate of matured cell is a very useful technique with a great interest recently. This approach can eliminate the drawbacks of direct usage of stem cells and allow the patient specific treatment in regenerative medicine. Overexpression of diverse factors such as general reprogramming factors or lineage specific transcription factors can change the fate of already differentiated cells. On the other hand, biomaterials can provide physical and topographical cues or biochemical cues on cells, which can dictate or significantly affect the differentiation of stem cells. The role of biomaterials on direct reprogramming has not been elucidated much, but will be potentially significant to improve the efficiency or specificity of direct reprogramming. In this review, the strategies for general direct reprogramming and biomaterials-guided stem cell differentiation are summarized with the addition of the up-to-date progress on biomaterials for direct reprogramming.

## Background

Regenerative medicine has been getting the spotlight in the medical science as a solution of intractable diseases. Especially stem cell therapy has great potential to cure many injuries and diseases. Stem cells have the ability to continuously divide and differentiate into various kinds of cells or tissues [1]. The main types of stem cells are embryonic stem cell (ESC), adult stem cell (ASC), and induced pluripotent stem cell (iPSC). ESC is derived from the inner cell mass of a blastocyst. It has pluripotency to be expanded unlimitedly and can differentiate to all three germ layers. But it is hard to get ESC and furthermore there is a severe ethical issue [2]. On the other hand, ASC, also called somatic stem cell, comes from the body after embryonic development, such as bone marrow, umbilical cord, adipose tissue, and blood cell. The source of ASC is more affordable than ESC, and ASC have less ethical issues compared to ESC [3, 4]. However, ASC is multipotent, not pluripotent, so the differentiation ability is less than ESC [5]. For overcoming the limitation of ESC and ASC, iPSC has been developed. iPSC is reprogrammed human cell by some defined factors to generate the patient-specific pluripotent cell lines [6, 7]. Yamanaka showed that iPSC can be generated using only four transcription factors, Oct4, Sox2, Klf4, and c-Myc [7]. iPSC can be obtained easily and has pluripotency to differentiate into any one of three germ layers, meaning that iPSC is a powerful regenerative medicine tool right away. However, iPSC also has several obstacles for practical applications. First of all, iPSC is not safe for clinical applications in its current state [8, 9]. Commonly, viral vector systems are used to generate iPSC, which might integrate into the host DNAs. More importantly, iPSC has risk to form tumors when transplanted in vivo because of the use of oncogene in the reprogramming process. Also, the efficiency of generating iPSC has been too low yet.

Direct reprogramming is a new approach to overcome diverse problems of stem cell therapies. Direct reprogramming means that reprogramming the somatic cell into a desired patient specific cell directly without passing through the pluripotent stem cell stage [10]. This method has a low risk about epigenetic remodeling and tumor formation. Also, it is more efficient and can be accomplished in an economy of time. In this review, direct reprogramming into various cell lineages will be introduced. Also biomaterials for affecting stem cell differentiation will be presented, and finally biomaterials to increase the efficiency of direct reprogramming will be introduced. Generally, direct reprogramming is also called transdifferentiation. Direct reprogramming and transdifferentiation are usually used as the same meaning, but exactly, direct reprogramming means the changing fate of somatic cell without dedifferentiation process and transdifferentiation means that less differentiated cell of certain lineage differentiates into other cell of similar lineage [11]. Here, the term ‘direct reprogramming’ will be used as the same meaning with transdifferentiation.

### Direct reprogramming

The general strategy for direct reprogramming uses transcription factors depending on the lineage of target. Most common cell source is the fibroblast from mouse or human. Here, recent examples of direct reprogramming will be discussed according to the final target cell type: Neural cells, cardiomyocytes and hepatocytes.

#### Direct reprogramming to neural cells

Neurodegenerative disorders, such as Alzheimers disease, Parkinsons’s disease and Huntingtons’ disease, have high lethality but there is no obvious cause and no effective medical treatment. Common symptoms of neurodegenerative disorders are continuously dying neural cells through necrosis or apoptosis, so cell regeneration of neural cells are necessary to cure those diseases. Thus, direct reprogramming approach can provide powerful regenerative therapies for neurodegenerative disorders [12].

A progenitor cell is undifferentiated state into mature functional cell, so it can differentiate into some types of mature cell but not all types of cell because it is not a stem cell. Different from direct reprogrammed neurons, direct reprogrammed neural progenitors can expand in vitro and possess the ability to differentiate into multiple neuronal subtypes. Mouse embryonic fibroblast can be reprogrammed to diverse neural progenitor cells. First, induced neural progenitor cells (iNPCs) are generated using forced expression of Yamanaka factors (Oct4, Sox2, Klf4, c-Myc) [13]. Although using the same factors which made iPSC, this group manipulated the specific signals affecting cell fates. This research also provided the possibilities of various applications with subtle signal changing because they used general reprogramming factors, not lineage specific transcription factors. Functional midbrain dopaminergic induced neuronal progenitors (iDPs) could be reprogrammed from mouse embryonic fibroblast or adult tail-tip fibroblast using 4 Yamanaka factors, Shh, and FGF8 [14]. Morphogen for midbrain development promoted the reprogramming and inhibiting JAK-STAT pathway which enhanced the efficiency of the reprogramming process. Induced oligodendrocyte precursor cells (iOPCs) were generated from mouse and rat fibroblast using transcription factors Sox10, Olig2, Zfp536 [15]. iOPCs could differentiate into astrocytes and myelinating oligodendrocytes. Although the differentiation efficiency of iOPCs was lower than that of neonatal OPCs, iOPCs are valuable because primary human tissues are limited. There are similar researches using human fibroblast. S Andrew et al. [16] made human induced neural progenitor cells (hiNPs) with miR-9/9, miR-124, and Neurod2 from human neonatal foreskin and adult dermal fibroblast. Micro RNAs, miR-9/8, and miR-124 regulated the neuronal differentiation and SWI/SNF-like BAF chromatin-remodeling complex, important for neuronal function, so they could control the neuronal fate. Additional Ascl1 and Myt1l increased the reprogramming efficiency. C Maucksch et al. [17] also generated hiNPs from adult dermal fibroblast using Sox2, Pax6, and recombinant protein transduction. Shh helped the regulation of neuronal differentiation from iNPs into functional and fully matured neurons. Overall, it took less time to convert neural precursor cells to functional neurons than iPSCs. However, iNPs required additional steps for redifferentiation into functional neurons unlike induced neurons. And, the multipotency of iNPs can be a strength but also be a weakness because the higher multipotency might have a risk going to pluripotency stage than functional cell, and a lower efficiency to be mature cell.

There also exist some known transcription factors which are specifically dominant in functional neurons, but not progenitor cells. Recent researches showed the overexpression of these transcription factors can induce the specific functional cell types. Direct reprogramming with neural-specific transcription factors could do interlineage reprogramming not only fate switching within the major lineage. Vierbuchen et al. [18] cloned 19 genes that are definitely expressed in neural tissues for screening the neural fate inducing factors. They found the three gene combination of Ascl1, Brn2 and Myt1l. The genes were sufficient to reprogram mouse embryonic dermal and postnatal tail-tip fibroblast into induced neurons (iNs). Although Ascl1 alone could induce neural functions like action potential, but coinfection of Brn2 and Myt1l was necessary for neural conversion and maturation. Similarly, the same three gene combination has been used in other groups [19–21]. To solve the problem of heterogeneous population of iNs, other researchers used lineage-specific factors for generating functional neurons. One group changed mouse embryonic and adult tail-tip fibroblast into induced motor neurons (iMNs) with Ascl, Brn2, Myt1l, Lhx3, Hb9, Isl1, and Ngn2. Addition of motor neuron specific factors to basic neural fate inducing factors efficiently induced motor neurons [22]. iMNs present motor neuron characteristics such as electrophysiological actor or forming functional synapses with muscle. Ectopic expression of Ascl1, Nurr1, Lmx1a could change mouse prenatal and adult tail-tip fibroblast to induced dopaminergic neurons (iDAs) [23]. This genetic reprogramming removed the major gene expression of the original cells, and expressed the induced target cell related gene. The strengths of this iDAs are rapid processing and maintenance. The reprogramming was achieved only in 6 days after expression of the factors and it remained a stable neuronal state over time.

There are other reports using human fibroblast. Human fetal limb and postnatal foreskin were changed to human induced neurons (hiNs) using Ascl1, Brn2, Myt1l and NeuroD1 factors [24]. Without NeuroD1, basic helix-loop-helix transcription factor, hiNs became functionally immature. Compared to mouse iNs, hiNs needed a long time to develop the synaptic activities. Instead of Ascl1, miR-124 with Myt1l and Brn2 also could generate hiN from human adult dermal fibroblasts [25]. miR-124 is a microRNA, most abundant in the mammalian CNS and upregulated in neurons. miR-124 helped the reprogramming to neurons by inhibiting the nonneuronal gene expression post transcriptionally. Human induced motor neurons (hiMNs) could be generated with the combination of Ascl1, Brn2, Myt1l, Lhx3, Hb9, Isl1, Ngn2, and Neurod1 [22]. Also human induced dopaminergic neurons (hiDAs) from human prenatal lung and adult skin cell could be generated by Ascl1, Nurr1, and Lmx1a [23]. The other combination for hiDAs is Mash1, Ngn2, Sox2, Nurr1, and Pitx3. Using this combination, human fetal lung fibroblast changed into hiDAs [26]. Exogenous expression of the five transcription factors significantly influenced on their endogenous counterparts. When hiDAs were injected into the rat model of Parkinson’s disease, the rotational behavior was stabilized distinctly. Also in vivo transplanted hiDAs are less likely to form tumors, although lentiviral vector may have integrated into the genome and block tumor suppressor genes.

Direct reprogramming does not rely on pluripotent stage, which is prone to tumors in their undifferentiated state. So, it can overcome the weakness of stem cell therapies. Also the source for direct reprogramming is sufficient in humans including patients’ themselves. Therefore, direct reprogramming is very promising for neurodegenerative diseases (Table 1).

**Table 1:** Direct reprogramming into neural cells

Species	Starting cells		Target cells	Reprogramming factors	Gene delivery	Efficiency (%)	Reference
Mouse	Fibroblast	embryonic, adult (tail-tip)	iNPC	Oct4, Sox2, Klf4, c-Myc	Lentivirus	0.7	[13]
Mouse	Fibroblast	embryonic	iDP	Oct4, Sox2, Klf4, c-Myc, SHH, FGF8	Lentivirus	26	[14]
Mouse	Fibroblast	embryonic	iOPC	Sox10, Olig2, Zfp536	Lentivirus	25	[15]
Human	Fibroblast	neonatal (skin), adult (dermal)	hiNP	NeuD2, MiR-9, 124, Ascl1, Myt1l	Lentivirus	5	[16]
Human	Fibroblast	adult (dermal)	hiNP	Sox2, Pax6	Nonviral plasmid or Recombinant protein	0.05	[17]
Mouse	Fibroblast	embryonic, postnatal (tail-tip)	iN	Ascl1, Brn2, Myt1l	Lentivirus	19.5	[18]
Mouse	Fibroblast	embryonic	iN	Brn2, Ascl1, Myt1l	pmax vector	7.6	[19]
Mouse	Fibroblast	embryonic	iN	Ascl1, Brn2, Myt1l	Lentivirus	11.6~16.1	[20]
Mouse	Fibroblast	embryonic, adult (tail-tip)	iMN	Ascl1, Brn2, Mytl1, Lhx3, Hb9, Isl1, Ngn2	Retrovirus	5~10	[22]
Mouse	Fibroblast	prenatal, adult (tail-tip)	iDA	Ascl1, Nurr1, Lmx1a	Lentivirus	18	[23]
Human	Fibroblast	fetal (limb), postnatal (skin)	hiN	Brn2, Ascl1, Myt1l, NeuroD1	Lentivirus	17~21	[24]
Human	Fibroblast	adult (dermal)	hiN	MiR-124, Myt1l, Brn2	Lentivirus	1.5~2.9	[25]
Human	Fibroblast	embryonic	iMN	Ascl1, Brn2, Mytl1, Lhx3, Hb9, Isl1, Ngn2, NeuroD1	Retrovirus	-	[22]
Human	Fibroblast	prenatal (lung), adult (skin)	hiDA	Ascl1, Nurr1, Lmx1a	Lentivirus	3~6	[23]
Human	Fibroblast	fetal (lung)	hiDA	Mash1, Ngn2, Sox2, Nurr1, Pitx3	Lentivirus	1~2	[26]

#### Direct reprogramming to cardiomyocytes

Heart disease is caused by not only atherosclerosis and hypertension but also daily eating and life habits, so it usually occurs more in older ages [27]. But, there is also a congenital heart malformation due to the improper development of cardiomyocytes during embryogenesis. If heart disease occurs, the treatment is not easy because the postnatal cardiomyocytes do not have regenerative capacity. So, there exist many articles regarding stem cell based heart disease therapies. However, stem cell-derived cardiomyocytes have many problems such as functional heterogeneity, maturity, potential tumorigenicity, low survival, retention of delivered cells, and insufficient stem cell source [28]. Direct reprogramming could provide better solution. The heart is composed of cardiomyocytes, vascular cells, and cardiac fibroblasts. Among them, cardiac fibroblasts occupy over 50% [29]. Cardiac fibroblasts support the cardiac structure, secrete signals and form scar formation upon cardiac damages. Direct reprogramming from cardiac fibroblasts to cardiomyocytes have been tried.

M Ieda et al. [30] induced cardiomyocyte-like cells (iCMs) from mouse neonatal cardiac and tail-tip dermal fibroblasts. They used three developmental cardiac transcription factors, Gata4, Mef2c, and Tbx5. This combination generated iCMs rapidly and efficiently within 7 days after transduction although full maturation required more times. iCMs showed spontaneous contraction activity. But, they indicated that the oscillation frequency of tail-tip fibroblast derived iCMs was lower than that of cardiac fibroblast derived iCMs. The other group used the same Gata4, Mef2c, and Tbx5 factors, but mouse adult cardiac fibroblast was used as starting cells [31]. They confirmed the reprogrammed hearts showing good sarcromere formation and contractile potential. Also iCMs could couple with viable endogenous cardiomyocytes electrically. In vivo test showed iCMs injected mouse had functional improvement for all parameters. Additional Thymosin β4 with the combination improved cardiac function and decreased scar size in vivo. K song et al. [32] reprogrammed mouse adult cardiac and tail-tip fibroblast into iCMs using Gata4, Mef2c, Tbx5, and Hand2. Previous researches showed the efficiency of reprogramming using adult fibroblast was lower than using neonatal fibroblast. But this group improved the efficiency up to the quadruple using additional Hand2. It was a very valuable result because cardiac fibroblast is the most prevalent cell type in adult hearts. iCMs of this group showed a similar calcium transient pattern to neonatal ventricular cardiomyocytes than adults and it could couple with surrounding myocytes through gap junction. The heart post myocardial infarction reduction was also confirmed in vivo. There have been some researches using mouse embryonic fibroblast. One group reprogrammed iCMs from mouse embryo mesoderm using Gata4, Tbx5, and Baf60c [33]. Baf50c with Gata4 could initiate the ectopic cardiac gene expression. Another group, who started reprogramming from embryonic fibroblast, interestingly used Yamanaka factors. Ectopic expression of Oct4, Sox2, Klf4, and c-Myc could make cardiomyocytes [34]. In spite of using the same factors, they grew cells in feeder cell with LIF-removed cardioinductive media. Then, early cardiac programmed cell was made. Of course, an early cardiac programmed cell needs cytokine and chemically defined media for terminal differentiation. It takes 3 weeks to make iPSC using Yamanaka factor, but it took only 12 days for direct reprogramming to cardiomyocyte using Yamanaka factor. Another promising alternative strategy is using microRNA. Jayawardena et al. [35] induced iCMs from mouse neonatal and adult cardiac fibroblast using a combination of miRNAs, mirR-1, 133, 208, and 499. Small size single miRNA can target multiple gene expression pathways simultaneously. Using miRNAs increased the reprogramming efficiency and homogeneity of resulting cells because miRNA could pack multiple transcripts in the same delivery vectors.

There are some researches using human fibroblasts to make cardiomyocytes. One group induced cardiomyocytes (hiCMs) from human fetal heart and neonatal skin fibroblast by using Gata4, Mef2c, Tbx5, Essrg, and Mesp1 [36]. Additional Myocardin and Zfpm2 enhanced the reprogram efficiency, sacromere formation, calcium transients, and action potential. And, hiCMs reprogramming efficiency could be improved by TGF-β signaling. Other research used miRNAs for hiCMs. They made hiCMs from human neonatal foreskin and adult cardiac, dermal fibroblast with Gata4, Hand2, Tbx5, myocardin, miR-1, and miR-133 [37]. Cardiac transcription factors were used equally with mouse cells, but human cells were more resistant to the artificial processing. Moreover, adult had more stable epigenetic programs and resistance than neonatal. Therefore, additional regulatory factors were necessary and so they chose miRNAs.

In summary, for inducing cardiomyocytes from fibroblast, cardiac transcription factors are needed. Among them, the main factors are Gata4, Tbx5, and Mef2c. Gata4 binds to its target site in the genome, so it plays as a pioneer. Tbx5 is essential for heart beating function. Mef2c is important in cardiac morphogenesis and myogenesis [38]. Further study is necessary for clinical application because hiCMs are still functionally immature and reprogramming efficiency varies. However, it is a very attractive strategy for curing both congenital and acquired heart diseases and improving the quality of life.

#### Direct reprogramming to hepatocytes

Hepatocyte is a main cell type in the liver. The liver regulates lots of physiological processes in our body. Liver diseases, such as liver metabolic diseases or acute liver failure, are very severe death causing problems [39]. For the treatment of liver diseases, liver transplantation is the golden standard. But, due to the limited supply of liver donation, hepatocytes transplantation can be an alternative.

Sekiya et al. [40] screened hepatic fate inducing factors and found that Hnf4a, Foxa1, Foxa2, or Foxa3 can reprogram mouse fibroblast into hepatocyte-like cells (iHeps). Foxa2 was used for embryonic fibroblast, and Foxa3 was used for adult dermal skin fibroblast. The characteristics of iHeps such as gene expression or morphology and hepatic functions are similar to that of mature hepatocytes. When iHeps were transplanted into the mouse liver, they could repair hepatic defects. Another method for inducing functional iHeps is an ectopic expression of Gata4, Hnf1a, Foxa3, and inactivation of p19Arf [41]. This combination could make iHeps from mouse adult tail-tip fibroblast. Gata4 and Foxa3 acted as chromatin modification, and then Hnf1a stabilized the induced hepatic gene expression. p19Arf is an important component of the cellular senescence pathway and it inhibits iPSC reprogramming. So the inhibition of p19Arf could overcome the proliferative limitation.

Recently, the similar researches using human cell were studied. Huang et al. [42] generated human induced hepatocytes (hiHeps) from human fetal limb and adult dermal fibroblast with expression of Foxa3, Hnf1a, and Hnf4a. This hiHeps had mature hepatic functions like biliary excretion of drug compound or CYP enzyme activities. Compared to fetal limb derived hiHeps, adult dermal derived hiHeps showed a low hepatic conversion rate. Another group used human newborn fibroblast to make hiHeps with Oct4, Sox2, and Klf in medium containing established growth factors and small molecule CHIR [43]. The factors they used are some of Yamanaka factors, but it did not undergo the pluripotent state. Strictly speaking, it is not a definite direct reprogramming strategy because they induced multipotent progenitor (iMPC) and then redifferentiated into hepatocytes.

Overall, the engraftment efficiency of iHeps does not reach to that of adult hepatocytes and it takes a long time for post-transplant maturation. The level of direct reprogramming to hepatocytes is not suitable for clinical application yet. However, patient derived hiHeps could be used in regenerative medicine and disease modeling in liver diseases in near future [44] (Table 2).

**Table 2:** Direct reprogramming into cardiomyocytes & hepatocytes

Species	Starting cells		Target cells	Reprogramming factors	Gene delivery	Efficiency (%)	Reference
Mouse	Fibroblast	neonatal (cardiac)	iCM	Gata4, Mef2c, Tbx5	Retrovirus	10~15	[30]
Mouse	Fibroblast	adult (cardiac)	iCM	Gata4, Mef2c, Tbx5	Retrovirus	12	[31]
Mouse	Fibroblast	adult (cardiac, tail-tip)	iCM	Gata4, Mef2c, tbx5, Hand2	Retrovirus	9.2	[32]
Mouse	Fibroblast	embryonic	iCM	Gata4, Tbx5, Baf60c	CMV promoter	<10	[33]
Mouse	Fibroblast	embryonic	iCM	Oct4, Sox2, Klf4, c-Myc	Retrovirus	-	[34]
Mouse	Fibroblast	neonatal, Adult (cardiac, tail-tip)	iCM	miR-1,133,208,499	Lentivirus	1.5~7.8	[35]
Human	Fibroblast	fetal (heart), neonatal (skin)	hiCM	Gata4, Mef2c, Tbx5, Esrrg, Mesp1	Retrovirus	13	[36]
Human	Fibroblast	neonatal (foreskin), adult (cardiac, dermal)	hiCM	Gata4, Hand2, Tbx5, Myocardin, miR-1,133	Retrovirus	12~19	[37]
Mouse	Fibroblast	embryonic, adult (skin)	iHep	Hnf4a, Foxa1, Foxa2 or foxa3	Retrovirus	0.3	[40]
Mouse	Fibroblast	adult (tail-tip)	iHep	Gata4, Hnf1a, Foxa3, inactivation of p19Arf	Lentivirus	20	[41]
Human	Fibroblast	fetal (limb), adult (dermal)	hiHep	Foxa3, Hnf1a, Hnf4a	Lentivirus	10	[42]
Human	Fibroblast	newborn	hiHep	Oct4, Sox2, Klf4	Retrovirus	-	[43]

### Biomaterials strategies for effective cell fate change

**Fig. 1:**
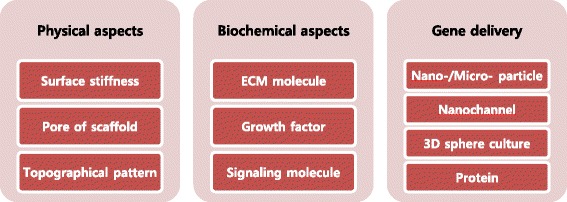
Classification of biomaterials strategies for cell fate

Biomaterials are synthetic or natural materials that can contact and integrate with biological system, but should not be harmful to the patient when performing intended functions [45]. A proper regulation of cell fate including stem cell or reprogramming of mature cell is the key issue in regenerative cell therapy. If reprogramming of cell is not controlled sufficiently, the therapy might cause teratoma, not treatment. There are numerous biomaterials for determining cell fate or helping the reprogramming process. Biomaterials can provide microenvironments mimicking a specific cell niche, allowing cells to differentiate into desired cell types [46]. The artificial stem cell niche can provide homing signals for attracting and localizing stem cells [47]. It may work similarly in direct reprogramming [48]. Classification of these biomaterials is not simple because one biomaterial can play multiple roles. Biomaterials can be classified in terms of physical properties such as surface, mechanical, electrical, and morphological properties. They can be classified in terms of the source such as natural, polymer, ceramic, and metals, or in terms of just shape such as 2D or 3D materials. In this review, we will describe the functions of biomaterials in terms of physical aspects, biochemical properties, and gene delivery (Fig. 1).

Biomaterials which regulate the stem cell fate can work in direct reprogramming also in the same principle. So, here some examples of biomaterials for stem cell differentiation and further recent researches about biomaterials for direct reprogramming will be described.

#### Physical aspects

Physical properties of biomaterials for cell reprogramming include mechanical strength (modulus) and surface topographies. First, the matrix elasticity of microenvironment can regulate the cell fate. Generally, stiffer substrates induce stiffer cells and softer substrates induce softer cells [49]. In the same vein, soft matrix can stimulate the differentiation of mesenchymal stem cell into neural like cells. Moderate elasticity favors myogenic differentiation, and rigid matrix promotes osteogenic differentiation [50]. A pioneering work of Engler A et al. [51] demonstrated the whole range differentiation of human mesenchymal stem cell by modulating the matrix elasticity, including neurogenic differentiation from elastic modulus in 0.1–1 kPa (brain), myogenic differentiation from elastic modulus in 8–17 kPa (muscle), and osteogenic differentiation from elastic modulus in 25–40 kPa (osteoids). Cell morphology also changed accordingly. So, the matrix properties can be a potent differentiation cue for mesenchymal stem cells [52, 53]. Similarly, physical signals like stiffness at the interface between cell and substrate can be a potent regulator for cell fate change. Soft hydrogel played an important role in making iPSCs via activation of mesenchymal to epithelial transition [54].

Fiber diameter of substrates also could influence on cell fate change [55, 56]. The fiber diameter of laminin coated electrospun polyethersulfone mesh could regulate the neural stem cell differentiation and proliferation. Generally, as the fiber diameter decreased, the neural stem cell proliferation rate increased. Cell morphology in small fiber showed stretched and multi directional shape, whereas the large fiber showed extended form along a single axis due to the size restriction. Although the main signals such as retinoic acid, fetal bovine serum, growth factor, etc. were necessary for differentiation or proliferation, the fiber diameter could increase or decrease the efficiency distinctly. Similarly, the pores of scaffold are also important cue. Levenger et al. [57] made 3D porous biodegradable polymer scaffolds which could influence the cell orientation, spreading and tissue structures. 3D scaffolds itself could allow the reconstruction and mimicking the complexity of the stem cell niche. In addition, this group made pores in scaffold to promote cell differentiation and homogeneity. Poly(lactic-co-glycolic acid) (PLGA) for degradability and poly(L-lactic acid) (PLLA) for mechanical stiffness were blended, and 250–500 μm pores were made. This scaffold helped the embryonic stem cell differentiation into desired cell with each suitable growth factor such as TGF-β for cartilage, Activin-A for liver like cells, and RA for neuroectodermal like structures. Another group made macroporous alginate scaffolds bearing cell adhesion peptide RGD and heparin binding peptide [58]. Combination of these peptides could provide different signaling processes in ECM-cell interactions and appropriate niche for cardiac stem cell behaviors.

Using a topographically patterned substrate without using potentially harmful chemicals for determining the lineage of stem cell is a safe and promising technique [59]. Surface topography with micropatterned nanoridge induced mesenchymal stem cell to acquire neuronal characteristics [60]. Compared to fibroblast like morphology of cells in smooth surface, stem cells became elongated morphology in ridge patterned hydrogenated amorphous carbon film. In other case, pattern of fibronectin on a coverslip generated by microcontact printing changed the differentiation tendency in accordance with the position [61]. Without pattern, human mesenchymal stem cell tends to differentiate into the osteogenic lineage at the edge, and the cells in the center tends to differentiate into adipocytes. However, cells cultured on the patterned coverslip changed the location of osteogenic versus adipogenic differentiation. Nanotopography also induced the changes in stem cell behaviors. Human mesenchymal stem cell on nanopatterned substrate showed lower integrin expression and FAK expression levels than unpatterned substrate in the cases of both soft (PDMS) and stiff (tissue culture polystyrene (TCPS)) substrate [62], meaning that nanotopographical cue has a more significant influence than the substrate stiffness on stem cell behaviors. Also, human mesenchymal stem cells on PDMS with nanograting were differentiated into neuronal like cells [63], proving that nanotopography can make stem cells to differentiate into specific, non-default pathways. In other study, hydrogen terminated ultra nanocrystalline diamond films could promote neural stem cell extension and protrusion [64]. There are some reports of using substrate topography in direct reprogramming. Direct reprogramming occurs in conjunction with significant changes on epigenome including the histone modification [65]. Leong et al. [20] reported the effect of substrate topography on direct reprogramming from fibroblasts to induced neurons. They demonstrated that cell-topography interactions can strongly influence the gene expression, neurite branching and outgrowth, and this shape and function determine the function of cells. In the course of neuronal induction, the cytoskeleton rearrangement is a crucial step. This cytoskeleton rearrangement responded to the substrate topography and substrate stiffness [66]. In other case, microtopography on cell adhesive substrate induced the change of cell morphology responsible for the modulation of epigenetic state [67]. Related to this result, micro- or nano-scale topography on PDMS induced pronounced changes in histone acetylation and methylation patterns and significantly promoted a mesenchymal to epithelial transition in adult fibroblasts [68, 69]. Specifically, the flat, microgrooved, and nanogrooved substates worked differently in terms of cell alignment. One group compared this by manufacturing the patterns using UV-assisted capillary force lithography and analyzed the direct reprogramming of somatic fibroblasts to neurons [70]. These passive topographical cues could offer a simple and effective possibility to enhance the efficiency of cell reprogramming.

Microparticle is another example of biomaterials working as a physical means. Microparticles have been widely used for encapsulation and releasing diverse molecules in biomaterial experiments [71, 72]. M Andres et al. [73] made various kinds of microparticles using various materials such as PLGA, agarose, and gelatin. These microparticles were stably integrated within aggregates of embryonic stem cells in a dose dependent manner. The presence of microparticles within stem cell aggregates could make stem cells to adopt specific differentiated phenotypes according to fabricated biomaterials. But, the efficiency of simple mixing of microparticles and stem cells was very low, so the forced aggregation was necessary. In almost all cases, maximal production of desired differentiated cells was shown when physical cue and chemical stimulus were applied together. Also, specific ligand-receptor interactions of growth factor or matrix molecules were more important than physical cues for regulating cells. However, physical properties of cell culture environments could also serve as a key factor in determining the cellular function and fate [49].

#### Biochemical aspects

Biochemical cues such as extracellular matrix (ECM) molecules, growth factors, and other signaling are essential to control the fate of cells. Especially, ECM components and structures regulate cell fate through integrin mediated activation and downstream signaling events [74]. Collagen, laminin, fibronectin, and matrigel are major ECM components. 3D collagen gel was used for CNS stem cell differentiation into functional neuronal circuits [75]. Because neuronal stem cells are anchorage dependent and attach to a solid surface, those solid polymer scaffolds are critical for neural tissue engineering. However, neural cells do not adhere to synthetic hydrogel, so the modification of hydrogel with ECM proteins such as collagen is necessary to provide relevant recognition and biological cell adhesion. In contrast, stem cells entrapped in biologically derived collagen hydrogel could rapidly expand in serum free medium containing bFGF, and showed the development to neurons, astrocytes, and oligodendrocytes. S Battista et al. [76] studied the effect of matrix composition of 3D constructs on embryonic stem cell differentiation. They made semi interpenetrating polymer networks, composed of diverse concentration of collagen, fibronectin, and laminin. In different compositions, embryonic body development and formation were different depending on the collagen concentrations. A high concentration of collagen inhibited cellular apoptosis so disrupted the embryonic body formation, and fibronectin accelerated the endothelial cell differentiation and vascularization. Laminin stimulated cardiomyocyte differentiation from embryonic stem cells. Collagen based hydrogel could also be applied in direct reprogramming. Endothelial progenitor cells were reprogrammed to smooth muscle cells, and this induced smooth muscle cell assembled blood vessel structure in a 3D dense collagen gel [77].

Fibrin is a large non globular protein formed by polymerization of fibrinogen and thrombin [78]. An ideal scaffold should have a relevant cell adhesion site and signals for promoting cellular differentiation. fibrin scaffolds with various concentrations of fibrinogen, thrombin, and aprotinin were characterized to make proper environments for neurogenesis [79]. They found out fibrinogen concentration is more important than thrombin concentration and higher thrombin concentration inhibits cell migration inside the fibrin scaffold. Also, when cell seeding density was high, more cell growth and differentiation were observed. Additional aprotinin could control the rate of gel degradation, so the optimal concentration of aprotinin was different depending on other compositions. Another group used fibrin gel for embryonic stem cell culture [80]. To retard the degradation of fibrin gel, fibrin gel was modified with poly(ethylene glycol) (PEG) as a secondary crosslinking method. This PEGylated fibrin culture increased the efficiency of cell expansion and modulated the differentiation of embryonic stem cells. PEG based hydrogel itself also has a potential for effective cell fate reprogramming. PEG prevents unwanted adsorption of proteins, which can induce altering cell signaling [81]. This property of PEG could potentially provide better environment for cell reprogramming, and it nearly doubled efficiency of making cardiomyocyte-like cells from fibroblasts [82]. This result suggests the future direction for developing a fully synthetic reprogramming microenvironment by adding some adhesion molecules or peptide to PEG based hydrogel. In this vein, one group successfully induced vessel like structures using human umbilical cord blood derived endothelial progenitor cells (EPCs) which can be easily isolated from the peripheral blood of adult or umbilical cord blood [83]. They designed PEG conjugated to bioactive peptides that provide cell adhesive and proteolytically degradable cues [84]. Combining the differentiation potential of EPCs and the characteristics of PEG hydrogel created the endogenous matrices like environments and formed 3D microvessel networks without supplemental angiogenic growth factors. Some group showed the affinity and density of ligand-receptor interactions at the biomaterial interface can be modified to direct cell fates [85]. Fibronectin coated or cycRGD presenting monolayer promoted osteogenesis. A high density linRGD stimulated myogenesis whereas a low density linRGD surface promoted neurogenesis. Also, high affinity ligands enhanced osteogenesis while low affinity ligands primarily enhanced myogenesis at a high density and neurogenesis at a low density. In this way, regulating affinity and density of ligand could direct certain stem cell differentiation. Furthermore, matrigel also useful as a cell culture scaffold because it presents appropriate extracellular biochemical cues for stem cell differentiation [57, 86].

Growth factor is an important biochemical cue, too. None of the growth factors can directly change cell fate exclusively to one cell type, but each growth factor can induce some lineage of cell fate. For example, Activin-A or TGFβ1 mainly induces mesodermal cells, bFGF, BMP-4, EGF, or retinoic acid induces ectodermal and mesodermal differentiation, whereas NGF and HGF allow all three embryonic germ layer differentiations [87]. Willerth et al. [88] applied combination of growth factors on 3D fibrin culture to determine their effects on embryonic stem cell differentiation. They used five different growth factors and found out the optimal concentration of each factors for inducing neurons and oligodendrocytes. The notch signaling pathway is also a key regulator of epithelial differentiation. Jagged-1 which is notch ligand on the biomaterial surface could regulate the stem cell differentiation [89]. Polystyrene or poly(hydroxyethyl methacrylate, HEMA) bound notch ligand could upregulate both epithelial differentiation and tight clustering. But, the presence of Jagged-1 as a soluble form could not act as an antagonist. So, it must be present as a bound form on other biomaterial surfaces. Other recent research suggested notch signaling biomaterials function in a time specific activation tunable manner [90]. This notch activation on embryonic stem cell promoted the ectodermal gene expression, and oriented Jagged-1 surface induced cardiac differentiation from cardiovascular progenitor cells.

Recent studies reported that, instead of using the transcription factors in direct reprogramming, the use of a cocktail of small chemical molecules only could change the cell fate. Small molecules are cell permeable, non-immunogenic, and cost-effective [91]. Once small molecules for direct reprogramming are selected throughout the screening, the addition of the cocktail of small molecules to the cell culture medium simply induced the change of cell fate. For examples, Forskolin, ISX9, CHIR99021, and SB431542 could induce functional neurons from mouse fibroblasts [92]. Nan et al. [93] chemically induced functional cardiomyocytes from human fibroblasts by a combination of nine compounds.

Although the categories were divided into physical and biochemical aspects in this article, the combination of physical and biochemical cues such as regulating both matrix stiffness and protein concentration on substrate together [94, 95] in stem cell studies is an effective and necessary strategy to change the fate of matured cells more efficiently.

#### Biomaterials for gene delivery

Gene delivery is a widely used technique in cell related research. It is an essential process for artificial changing of cell fate or regulation of stem cell differentiation. Before the development of new method of gene delivery, viral vector had usually been used. But, this viral vector has safety problems because viral vector may interfere with the host sequence randomly. Therefore, the development of safe gene delivery methods was necessary.

First alternative method developed was using nanoparticles for gene delivery. For example, stimuli responsive Hyaluronic acid (HA) -ss- Polyethylenimine (PEI) nanoparticles were developed as a non-viral gene vector [96]. Positively charged PEI can form nanocomplex with the negative charge of DNA, and release it in the endosome, but limited by its cytotoxicity. Many tissues have HA receptors, and HA has polyanionic characteristic that prevents the non-specific interaction between serum and PEI, so HA can provide the stability and specificity of the particle. Disulfide bond can give the enhanced release of DNA release in the endosome. Similarly, Zhao et al. [97] made Chitosan (CS) -ss-PEI nanoparticles for mediating osteogenic differentiation. In other case, magnetic nanoparticles based vector system were applied for safe and efficient gene delivery [98]. Magnetic nanoparticles covered by a biocompatible surface coating were functionalized for delivery of therapeutic biomolecules such as DNA, siRNA, or shRNA. Similarly, a micro/nanochannel array based electroporation system was suggested [99–101]. This is a single cell transfection system which can deliver charged agents directly into the cytosol electophoreitcally, so it is possible to control the precise dosage. All of these transient gene expression using non-viral vector can prevent the problems with overexpression of genes and interference of viral vectors.

Other safe replacement method of viral vector mediated gene delivery is using protein itself. But half-life of protein is short, so it requires a large dose and high cost. For example, D Kim et al. [102] used reprogramming proteins fused with a cell penetrating peptide (CPP). This method can avoid the genetic integration and immunogenic problems, but the efficiency was not good. Generally, the efficiency of protein delivery into the cell is very low because protein is macromolecule and hard to penetrate cell membranes. Thus, there is a strong need for biomaterials to increase the efficiency of intracellular delivery of proteins. Qutachi et al. [103] delivered proteins into embryoinic body using PLGA microparticles to control the differentiation of embryonic stem cells. Also, Bian et al. [104] made nanoparticles, which can load growth factor proteins and combine with hydrogel scaffolds. They could induce chondrogenic lineage from mesenchymal stem cell by the protein loaded microsphere scaffolds.

Lastly, 3D sphere culture is a promising method for the safe and efficient gene deliver. Neural crest associated cells were induced by culturing fibroblasts with a single factor FOXD3 without viral vector on the chitosan substrate instead of TCPS [105]. Then, the cells grew in a spheroid shape and held together the plasmid. Actually, the result showed a quite low transduction efficiency, but a high viability (~100%) after transfection. Similarly, 3D sphere induced neural progenitor like cells from fibroblast were reported [106, 107]. Although the repeated transduction is necessary due to the low transduction efficiency, it can be free form the potential risk of viral vector such as gene disruption, instability, and tumor formation [108].

Still, there is no perfect biomaterial which fulfills all requirements such as biocompatibility, biodegradability, and high efficiency. However, one can select among available materials and develop new materials for each purpose based on previous studies.

## Conclusions

In summary, direct reprogramming is a promising medical technique for degenerative disease, incurable disease, disease modeling, and drug development. Moreover, biomaterials have the potential to empower this technique to be more effective and stable. However, there are lots of problems to overcome furthermore. In the aspect of direct reprogramming, the way of interlinage conversion is not established yet. Also, the safety is not proved in longer term clinical trials, and the efficiency of direct reprogramming is still too low to produce enough amount of cells for clinical treatment. Heterogeneity and immaturity of conversed cells also should be improved. In the aspect of biomaterials, a fully synthetic reprogramming microenvironment needs to be developed for quality control and dealing with immunogenic problems. Also, there is no clear evidence or report that biomaterials can greatly improve the efficiency of direct reprogramming so far. Overall, the more effective combination of biochemical cue and physical cues for each direct reprogramming process must be set into shape. And, the issues of time consuming, low efficiency, and treatment convenience should be considered together for clinical application.

## Abbreviations

ACS: Adult stem cell
CPP: Cell penetrating peptide
CS: Chitosan
ECM: Extracellular matrix
EPCs: Endothelial progenitor cells
ESC: Embryonic stem cell
HA: Hyaluronic acid
HEMA: Poly(hydroxyethyl methacrylate)
hiCMs: Human induced cardiomyocytes
hiDAs: Human induced dopaminergic neurons
hiHeps: Human induced hepatocytes
hiMNs: Human induced motor neurons
hiNPs: Human induced neural progenitor cells
hiNs: Human induced neurons
iCMs: Induced cardiomyocyte-like cells
iDAs: Induced dopaminergic neurons
iDPs: Dopaminergic induced neuronal progenitors
iHeps: Induced hepatocyte-like cells
iMNs: Induced motor neurons
iMPCs: Induced multipotent progenitors
iNPCs: Induced neural progenitor cells
iNs: Induced neruons
iOPCs: Induced oligodendrocyte precursor cells
iPSC: Induced pluripotent stem cell
PDMS: Polydimethylsiloxane
PEG: Poly(ethylene glycol)
PEI: Polyethylenimine
PLGA: Poly(lactic-co-glycolic acid)
PLLA: Poly(L-lactic acid)
TCPS: Tissue culture polystyrene
